# Formatt: Correcting protein multiple structural alignments by incorporating sequence alignment

**DOI:** 10.1186/1471-2105-13-259

**Published:** 2012-10-06

**Authors:** Noah M Daniels, Shilpa Nadimpalli, Lenore J Cowen

**Affiliations:** 1 Department of Computer Science, Tufts University, 161 College Ave, Medford, 02155, MA, USA; 2 Department of Computer Science, Princeton University, 35 Olden St, Princeton, 08540, NJ, USA

## Abstract

**Background:**

The quality of multiple protein structure alignments are usually computed and assessed based on geometric functions of the coordinates of the backbone atoms from the protein chains. These purely geometric methods do not utilize directly protein sequence similarity, and in fact, determining the proper way to incorporate sequence similarity measures into the construction and assessment of protein multiple structure alignments has proved surprisingly difficult.

**Results:**

We present Formatt, a multiple structure alignment based on the Matt purely geometric multiple structure alignment program, that also takes into account sequence similarity when constructing alignments. We show that Formatt outperforms Matt and other popular structure alignment programs on the popular HOMSTRAD benchmark. For the SABMark twilight zone benchmark set that captures more remote homology, Formatt and Matt outperform other programs; depending on choice of embedded sequence aligner, Formatt produces either better sequence and structural alignments with a smaller core size than Matt, or similarly sized alignments with better sequence similarity, for a small cost in average RMSD.

**Conclusions:**

Considering sequence information as well as purely geometric information seems to improve quality of multiple structure alignments, though defining what constitutes the best alignment when sequence and structural measures would suggest different alignments remains a difficult open question.

## Background

Researchers in protein biology must often build structural alignments of multiple homologous proteins. Generally, both the protein sequence and its 3D structure are available to a structural alignment program. The structural alignment program typically produces both a rigid body transformation that aligns the structures in space, plus a sequence alignment derived from that structural alignment that proposes homologous residue-residue correspondences. For a recent survey of the best current structural alignment programs available, see [1]. In the absence of hand-curated gold-standard benchmarks, the quality of protein structure alignment is usually measured based on purely geometric measures: some function of the number of residues declared to be alignable, together with an average RMSD score for aligned residues, plus perhaps a penalty for gaps. Similarly, most of the best structural alignment programs in use today begin by ignoring all sequence information, and working only with the geometric location of the *C*_*α*_ atoms of the protein backbones. It seems that this extra information could be used to improve protein structural alignment. However, a meaningful way to incorporate sequence information into structural alignment algorithms in order to improve their performance has remained elusive.

One of the reasons it has not been clear how best to incorporate sequence information into structural alignment programs is that it is unclear what the goal is, or rather, the goal might be problem-dependent. When a sequence alignment and a structure alignment of two protein sequences give different answers, which one is correct? If the correct alignment is defined solely based on the geometric location of the *C*_*α*_ atoms of the protein backbones, then this alignment can always be computed without ever looking at the protein sequences. At the opposite end of the spectrum, we could imagine a “true” correct alignment to be one that aligns residues that have evolved from residues in a common ancestor protein. Ignoring the fact that constructing a gold-standard benchmark to test alignment algorithms according to this standard is impossible without knowing ground truth, such an alignment might result in aligned regions with very little geometric similarity, since there are known examples of proteins with high sequence similarity but markedly different folds [2].

Several researchers have developed algorithms, including 3DCoffee [3], PROMALS3D [4], and SALIGN [5], that consider both sequence and structure when constructing protein alignments. As has been demonstrated by Kim and Lee [6], structure-based methods produce better sequence alignments than methods based on sequence information alone. These algorithms have all, to some extent, had to address the question of what their hybrid algorithm considers a “correct” alignment. However, with the notable exception of SALIGN (see below) most of these papers try to use *structural* information to improve *sequence* alignments, whereas the goal of this paper is to use *sequence* information to improve *structural* alignments. Even though the “correct” alignment in both scenarios is presumably the same, these are two very different problems, because the natural assumptions on the inputs to the two problems are completely different: i.e., sequence alignment programs cannot assume structural information is available for all proteins.

**Figure 1:**
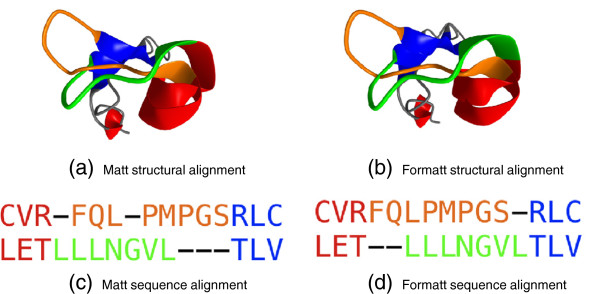
**Formatt frame-offset repair example.** Example of Formatt’s frame-offset repair on a subset (residues 37-50 of chain A of PDB ID 1c9f, and residues 64-76 of chain A of PDB ID 1d4b) of the HOMSTRAD “CIDE-N” group. In both sequence and structural alignments, difference between Matt and Formatt are shown in orange and green; red and blue regions are *α*and *β*structures aligned identically by Matt and Formatt. Note that the Formatt alignment has fewer non-core residues (three) than Matt (five).

Instead of asking if (partial) structural information can help sequence alignment algorithms, this paper instead focuses on what we believe is a substantially easier computational problem: we ask if sequence information can help structural alignment algorithms in the typical setting where purely structural alignment algorithms are employed, specifically when 3D structural information is available for all the proteins in the set. We suspected it would help, because anecdotally, for even the best structural alignment programs, we knew there were always cases where it seemed a human being could hand-“correct” the alignment into something that made more sense from a sequence point of view, with little or no loss in geometric fidelity. The kinds of errors produced by structure alignment programs that do not take sequence into account can be illustrated by an example pair of proteins, aligned by our group’s own structure alignment program, Matt [7]. Figure 1 illustrates how the structural alignments produced are quite similar, but the Formatt sequence alignment has fewer gaps, and thus fewer non-core residues (three) than Matt (five). The HOMSTRAD gold-standard alignment for these chains (PDB IDs 1c9f:A residues 1-87 and 1d4b:A residues 1-122) indicates only one gap in this short region. In this instance, Formatt more closely matches HOMSTRAD both within this short region and for the alignment as a whole. Note that while we have chosen to show a bad alignment produced by our Matt program, all the other purely structural alignment algorithms that we have tested will sometimes produce similar types of errors. 

To avoid these offset problems, we modify Matt to also take into account sequence similarity, in order to correct this type of register error. In particular, we introduce “Formatt” which stands for “Frame Offset Repair Matt” which uses the same geometric information that Matt uses to decide what regions of the protein should be considered alignable. Formatt allows Matt to construct its bent alignment, which breaks a protein up into small tightly aligned blocks, between which are regions where Matt would greedily align the backbone between blocks (the Matt “extension phase”) using solely geometric criteria. Formatt, by contrast, considers both geometric and sequence similarity criteria in choosing which residues to align in these regions.

Note that our Matt structural aligner is specifically optimized for more distant homology [8] and as we find again in this paper, classical aligners may perform better on highly homologous sequences. However, the hope is the Formatt correction will improve Matt performance on closely homologous sequences while preserving Matt’s performance advantage on remote homologs. We show below that this is indeed the case.

We test the performance of Formatt against the original Matt [7], against Mustang [9], another well-known multiple structure alignment program, and against SALIGN [5], which like Formatt incorporates sequence information into a structural alignment. We also considered 3DCoffee [10] and Promals3D [4]. We were unable to run 3DCoffee successfully on more than a small sample of the HOMSTRAD benchmark, because it was incompatible with our network environment and crashed our fileserver repeatedly, but found on that sample that it was not competitive. We also found Promals3D not to be competitive, but we were able to run it successfully and report results in Tables 1 and 2. Note that Promals3D does not provide a 3D structural alignment, so for Promals3D we can only report sequence-based measures of alignment quality. Of course, as remarked above, to be fair to 3DCoffee and Promals3D, they can also produce alignments (which Formatt cannot) when structural information is only available for a subset of the protein sequences to be aligned, and were not optimized for the full-information structural alignment problem. 

**Table 1:** HOMSTRAD multiple alignments (all values are averages)

	**Core length**	**RMSD**	**Seq**	**Str**	**Cons**	**Partial length**	**Partial Seq**	**% Correct**
HOMSTRAD	126.8	2.71	1.37	1.82	1.60	136.2	2.13	(100%)
Mustang	152.8	3.60	1.54	1.86	1.70	165.3	2.26	**79.3**%
Matt	**178.4**	1.72	1.54	1.55	1.55	189.0	2.34	73.4%
SAlign	172.6	2.29	1.79	2.86	2.32	**190.8**	2.45	78.1%
(Promals3D)	***186.8***	-	1.55	-	-	***198.6***	***2.08***	43%
Formatt (mafft)	148.2	**1.46**	**1.36**	**1.35**	**1.35**	155.6	2.18	78.7%
Formatt (clustalw)	169.3	1.72	1.47	1.55	1.51	182.0	**2.11**	72.9%
Formatt (muscle)	169.6	1.72	1.48	1.55	1.51	179.1	2.15	72.4%
Formatt (probcons)	168.9	1.73	1.50	1.55	1.53	178.8	2.18	73.6%

**Table 2:** SABMark Twilight Zone multiple alignments (all values are averages)

	**Core length**	**RMSD**	**Seq**	**Str**	**Cons**	**Partial length**	**Partial Seq**
Mustang	63.4	4.12	3.92	3.05	3.49	97.6	4.46
Matt	**66.9**	2.64	4.15	2.29	3.35	84.0	4.58
SAlign	59.6	3.51	3.88	2.83	3.36	**90.7**	4.34
(Promals3D)	***75.4***	-	3.89	-	-	***111.8***	4.39
Formatt (mafft)	45.01	**1.97**	**3.85**	**1.77**	**2.81**	54.0	**4.32**
Formatt (clustalw)	64.05	2.75	3.98	2.32	3.15	76.2	4.49
Formatt (muscle)	64.37	2.77	3.99	2.33	3.16	76.0	4.55
Formatt (probcons)	64.5	2.97	4.00	2.44	3.22	75.6	4.54

The metrics under which we tested performance on HOMSTRAD include the correct gold-standard reference alignments (which were curated by hand). On the SABMark “Twilight Zone” benchmark [11], which we chose to capture the alignment of more remotely homologous proteins, there is no gold-standard reference, and so another measure of alignment quality must be devised. We show that Formatt alignments are superior to Matt alignments according to a purely objective measure that does not require a reference alignment; namely, the “Staccato” Seq, Str, and Cons scores as introduced by Shatsky, Nussinov and Wolfson [12]. While Mustang and SALIGN both produce reasonable HOMSTRAD alignments, and in fact their HOMSTRAD alignments match the reference alignments slightly better than either Matt or Formatt, neither Mustang nor SALIGN produce SABMark alignments with reasonable RMSD, in contrast to both Formatt and Matt.

Formatt source code is freely available for download under the Gnu Public License at http://bcb.cs.tufts.edu/formattwhere we also make available HOMSTRAD and SABMark benchmark reference alignments aligned by Formatt.

## Methods

### Matt

The Matt structural aligner [7] belongs to the class of fragment-pair chaining method aligners. Matt finds blocks of between 5 and 9 amino acids in each chain participating in a multiple alignment that share close spatial alignment, without regard to the fact that the regions between these blocks may include impossible bends, translations, or twists. Matt then extends these aligned blocks, adding adjacent amino acids that do not diverge greatly in spatial alignment. Thus, Matt aligns protein sequences based on root mean square distance (RMSD). Ultimately, Matt chooses an optimal alignment based on a balance of RMSD and the number of aligned residues. Clearly, at the extrema, an RMSD of 0 could be found for any set of amino acid chains with a length of only 1 residue; likewise, maximally long alignments could be achieved without regard to RMSD. Matt solves a bi-criterion optimization problem, balancing the length of the aligned cores with the minimization of RMSD. This balance was achieved by finding a linear combination of RMSD and core length that optimally separated SABmark [11] positive from decoy chains at the superfamily level of homology.

### Improving upon Matt

The chief limitation of Matt’s approach is that the regions in between the original, closely-aligned, 5-9 amino acid blocks are still aligned purely according to this balance between core length and RMSD, and thus the final alignment may choose arbitrarily between different possible alignments of similar RMSD values. This can lead to otherwise obvious sequence similarities being discarded due to negligible differences in RMSD. By preserving sequence information, and allowing the input from a pure sequence alignment tool to influence the final alignment, we aim to improve the alignments of these regions between closely-aligned blocks.

Formatt produces an initial “bent” alignment of 5-9 amino acid blocks, identically to Matt. It then extends each aligned block as follows: given a region of residues between blocks, produce candidate alignments using a sequence aligner (of which Formatt supports CLUSTAL-W [13], MUSCLE [14], ProbCons [15] and MAFFT [16]), as well as a greedy structural alignment within an RMSD threshold of 5Å using the original Matt algorithm. Formatt then computes the Staccato [12] “Cons” conservation score for both the resulting sequence-based alignment and structural alignment, and chooses the alignment for this region based on the lower (better) conservation score. We describe our implementation of the Staccato score below.

We present results for Formatt based on all four sequence aligners, but considering our results in the next section, we clearly recommend MAFFT as the default sequence aligner for use with Formatt on closely homologous sequences.

### Core Alignments, Partial Alignments

Both Matt and Formatt support partial alignments; that is, they allow columns that align only some subset of structures, while other substructures have gaps in these positions. We define the *core* of the alignment to be the columns in which there are no gaps placed in the alignment; that is, every structure contributes a residue. When evaluating our alignments, some metrics make sense to evaluate over the entire alignments, while other metrics make sense to evaluate only on core positions: in particular, since the optimal structural superimposition based on a particular protein sequence alignment is classically computed based only on core positions, all measures with a geometric component are only defined based on core positions, namely RMSD, Staccato Str, and Staccato Cons.

In order to evaluate the effect of partial alignment, we report not only the aligned core length (the number of columns of the alignment in which every protein chain has a residue rather than a gap) but also a partial core length. We define partial core length as the total length of the alignment, *l*, multiplied by a partial alignment factor *p*, where *p* is the average, over the length of the alignment, of the number of possible pairs in each column in which both members contain residues, divided by the total number of possible pairs (which is simply *k* choose 2 for an alignment of *k* proteins): ∑i=0nmk2n, where *n* is the number of columns in the alignment and *m* is |{*i*,*j*|*i*,*j*←0⋯*k*∧*i*≠*j*}| such that neither *i* nor *j* are gaps. Similarly, we can report Staccato Seq scores for both core and partial alignments.

### Staccato scores

In order to determine whether sequence-based or structure-based alignment performs better for a given region of the multiple alignment, we implement the Staccato scores described by Shatsky et al. [12].

Given a multiple structure alignment *A* and columns *c*∈*A*, let the “Seq” sequence conservation score *Seq*=9×(1−(*Seq*^′^ + 4)/9*.*75), where ${\text{Seq}}^{\prime }=\frac{\underset{c\in A}{\sum }\overset{N}{\underset{i}{\sum }}\overset{N}{\underset{j>i}{\sum }}w_{i}w_{j}S(c_{i},c_{j})/W}{\left|A\right|}$ and $S(c_{i},c_{j})=\left\{\begin{array}{ll} \text{Blosum}62(i,j) & \;\text{if}\;i\neq j,\\ \overset{20}{\underset{i=1}{\sum }}\text{Blosum}62(i,i) & \;\;\text{otherwise}. \end{array}\right)$

Let the weights above be defined as $w_{i}=\frac{\overset{N}{\underset{j\neq i}{\sum }}d(i,j)}{\left(N-1\right)}$ and $W=\overset{N}{\underset{i}{\sum }}\overset{N}{\underset{j>i}{\sum }}w_{i}w_{j}$, where $d(i,j)=1-\frac{\text{PercentIdentity}(S_{i},S_{j})}{100}$. *N* is the number of sequences in the alignment *A*.

Also given an alignment A and columns *c*∈*A*, let the “Str” structural conservation score $\mathrm{Str}=\frac{\underset{c\in A}{\sum }D(c_{i},c_{j})}{\left|A\right|}$, where $D(i,j)=\frac{\underset{c\in A}{\sum }\left\{\begin{array}{ll} 9 & \;\;\text{if}\;\mathrm{rmsd}(c_{i},c_{j})>22.62\text{Å},\\ \frac{1}{f+\frac{1-f}{\mathrm{rmsd}(c_{i},c_{j})}} & \;\;\text{otherwise} \end{array}\right)}{\left|A\right|}$, and *f* is defined as 0.07 as it was in [12].

Finally, the overall Staccato “cons” conservation score is simply *ω*×*Seq* + (1−*ω*)×*Str*, where *ω* is set as 0.5, equally weighting the sequence and structure scores, just as in [12].

We diverge from the Staccato paper in the way that we compute these scores in one important respect: by default, we only consider core positions in the alignment (where a core position places no gaps in the alignment) when scoring a multiple alignment.

In addition, we also compute a partial Seq score identical to the Seq score in the Staccato paper, treating an alignment of a gap with any residue or with another gap as a score of zero. We report these values alongside the final conservation score.

### Validation

In order to quantitatively assess Formatt’s performance, we evaluate it against two well-known benchmark sets, HOMSTRAD [17] and SABMark [11].

The HOMSTRAD multiple-alignment benchmark consists of a manually curated set of 1,028 alignments, each of which contains between two and 41 structures. To duplicate the benchmark in [7], we test our methods on the 398 HOMSTRAD alignments with more than two structures in the alignment (that is, HOMSTRAD sets with between three and 41 structures that necessitate a multiple rather than a pairwise structure alignment program). For HOMSTRAD alignments, we can assume the manually curated alignment form a gold-standard set of “correct” alignments.

The SABMark benchmark is divided into superfamily and “Twilight Zone” benchmark datasets, each of which contains subsets of 3 to 25 remotely homologous protein structures. We test Formatt and its competitors on the 209 subsets in the “Twilight Zone” set. Note that for these more distant homologs, we do not have a gold-standard set of “correct” alignments, and must determine alignment quality by objective means, such as core length, average pairwise RMSD, as well as the Staccato scores, as introduced by [12] and discussed above.

## Results

As can be seen in Table 1, on the 398 HOMSTRAD multiple alignments, according to the Staccato “Cons” overall score, Formatt with MAFFT performs best of all the choices tested (including as compared to the supposed “gold-standard” hand-curated HOMSTRAD alignments). It produces the best RMSD, average sequence, and average structure scores compared to all the methods as well. In fact, it completely dominates the supposed HOMSTRAD gold-standard alignment on not only these measures, but also average core length, meaning it is producing longer alignments with better sequence and structural agreement than the gold-standard manually curated alignment. Note that Formatt (MAFFT) is, however, being more conservative with declaring residues in the common core of the alignment than any of the other alignment programs we tested (but still less conservative than the HOMSTRAD gold-standard alignment). The other versions of Formatt that we tested (with MUSCLE, CLUSTAL-W, and ProbCons as the internal sequence alignment program) have worse Staccato Seq, Str, and Cons scores than Formatt (MAFFT), and have a smaller percentage of their residues agreeing with the HOMSTRAD manually curated alignment. However, they place an average of about 20 more residues in the common core of the alignment. As can be seen in Table 1, the other versions of Formatt perform much more similarly to original Matt (the purely geometric version of our structural aligner). These versions of Formatt still have slightly shorter core sizes than original Matt alignments, but then achieve slightly better sequence alignment scores, with similar struct and RMSD scores.

Table 2 shows that the pattern is similar for the SABMark “Twilight Zone” benchmark set, though of course we do not have gold-standard reference alignments for SABMark, making the results harder to interpret. Again, however, Formatt (MAFFT) is the most conservative in terms of common core length, but then achieves the best sequence, structural, and combined conservation scores. However, here is where other choices of sequence aligner within Formatt might be desirable if a longer core length is the goal. In particular, if we examine Formatt (CLUSTAL-W), Formatt (MUSCLE) and Formatt (ProbCons), they, along with original Matt clearly outperform either Mustang or SALIGN on this benchmark, achieving better RMSD as well as Staccato Seq, Str, and Cons scores for alignments with longer core lengths. Which of the Matt or Formatt variants is best then becomes a question of how one wishes to trade off the importance of the sequence versus the structural scores.

## Discussion

**Figure 2:**
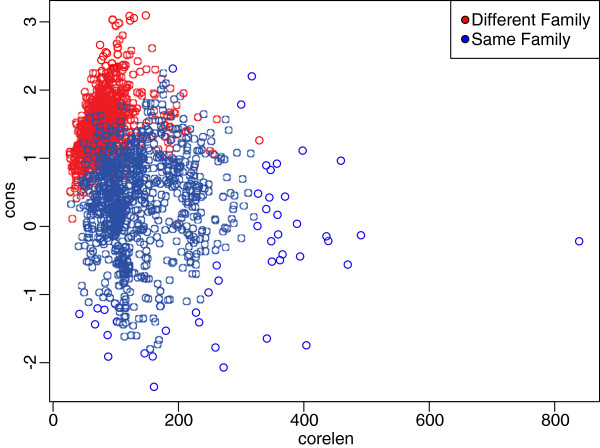
**Staccato conservation score vs. alignment length.** Separation of 1,000 domain pairs where both domains are in the same SCOP family, and 1,000 domain pairs where both domains are in different SCOP families, along the dimensions of Staccato conservation score and core length of the pairwise alignment.

In this implementation, we have followed the example of Shatsky *et al*[12] in equally weighting the sequence and structure components of the Staccato score, and we have left the choice of longer aligned core versus better alignment quality to the user. We are using the Staccato scores, but there are several weaknesses in this approach. First, the combined “Cons” score, which we use to decide if Formatt should use a sequence- or structure-based alignment for a particular region, equally weights the “Seq” and “Str” scores, but this seems arbitrary. Secondly, and more seriously, Staccato scores are not length-invariant – that is, while they are appropriate to compare different alignments of the same length, they will always prefer shorter alignments. In fact, one could worry that the only gain that Formatt makes over Matt in Staccato score is due to Formatt preferring shorter, more conservative alignments (particularly when Mafft is used as the sequence aligner). To show that this is not the case, we created a ‘truncated’ Matt alignment by ranking the columns of the Matt alignment by Staccato Cons score, and, on a structure-by-structure basis, greedily dropped columns from the Matt alignment until it matched the Formatt (Mafft) alignment in length. This resulted in an identical average core length of 148.2 on the HOMSTRAD and 45.01 on the SABMark benchmarks. However, Formatt (Mafft) is qualitatively better than this truncated Matt, both in terms of the Staccato Cons score (1.39 for truncated Matt versus 1.35 for Formatt (Mafft) on HOMSTRAD, and 2.86 for truncated Matt versus 2.81 for Formatt (Mafft) on SABMark) and in terms of the percent correct on HOMSTRAD (78.4% for truncated Matt versus 78.7% for Formatt (Mafft)). This proves that it is worth considering sequence alignment as Formatt does, directly, and not just in terms of Staccato score. The problem of how to normalize a Staccato measure of alignment ‘quality’ with alignment length remains an interesting question. One way to achieve this normalization is suggested by [8]. A plot of aligned core length versus Staccato conservation score for one thousand random pairs of same-family and different-family protein domains can illustrate a possible method for trading off between core length and alignment quality (see Figure 2). We see that an optimal linear separator of 0*.*126×*x*−0*.*213 divides same-family from different-family domains. Thus, given two possible alignments *a*_1_ and *a*_2_, with Staccato “cons” scores of *c*_1_and *c*_2_, and core lengths of *l*_1_ and *l*_2_ respectively, we could view these as points in the space defined by “cons” score and core length. We could then compute the y-intercept of a line with a slope of *.*0126 through each point; we would then favor the alignment with the lower y-intercept. We suggest that this would be a plausible way to rationally quantify the trade-off between alignment quality and core length. 

We remark that, while results are not shown in the tables above, we also tested Promals3D on the HOMSTRAD benchmark set. Note that Promals3D outputs only a sequence alignment without coordinates, so an RMSD or other structural scores were not calculated. However, when we compared the Promals3D to the HOMSTRAD gold-standard alignments, the average percentage correct was only 43%. We tested a subset of the HOMSTRAD benchmark set against 3DCoffee and the results were even worse. Thus, we conclude that Promals3D and 3DCoffee are not producing competitive alignments on this benchmark.

## Conclusions

We have introduced Formatt and showed that incorporating sequence information can improve the quality of structural alignments, both in terms of gold-standard alignment benchmarks, and in terms of objective measures of sequence and structural alignment quality such as the Staccato score [12]. We were particularly interested in “correcting” Matt structural alignments to better capture sequence homology because of our extensive use of the Matt structural alignment program in the training phase as we build HMMs [18] and Markov Random Fields [19,20] from sets of solved protein structures that fold into the similar shapes, to learn to recognize new protein sequences that match these models. More consistent alignments lead to better structural templates, and therefore better motif recognition programs. This is the same problem domain that motivated the work on the SALIGN program as well [5].

Formatt is a variant of the Matt [7] multiple structure alignment program, one of a new generation of structural alignment programs that incorporate flexibility into multiple protein structure alignments. Other recent pairwise and multiple structure alignment programs that also incorporate some form of flexibility into alignments include FlexProt [21], Fatcat [22], Posa [23], Rapido [24], and FlexSnap [25]. It would be interesting to see if some form of sequence alignment could be incorporated into these programs as well, and whether it could improve their structural alignments.

The question of what makes a good alignment is not simple to answer. Recall Figure 1, in which the greater number of core residues in the Formatt alignment seems intuitively better than the very slightly tighter RMSD of the Matt alignment. In reality, aligning proteins is an attempt to capture the evolutionary history by which those proteins diverged. However, since in general we do not have a history of every mutation in chronological order, we must rely on sequence and structure conservation scores, and infer that the closest alignment, representing the fewest changes, is the most likely. Why do we seek to more faithfully reconstruct evolutionary history in protein alignments? We commonly use protein alignments to build profiles for remote homology detection approaches such as profile hidden Markov models (HMMs) [18,26] or Markov random fields [19,20]. The match states of an HMM derive from the core positions of a multiple alignment, and the HMM is a probabilistic model which attempts to capture the evolutionary process by which proteins are related. Thus, alignments that more faithfully represent evolutionary relationships should lead to more accurate remote homology detection. An interesting, if computationally intensive measure of alignment quality would be to see whether Formatt alignments of protein superfamilies lead to more accurate HMM predictions of remote homology than do the comparable alignments produced by existing software such as Matt.

As mentioned above, we followed [12] in using a constant *ω* of 0.5 when weighting the Staccato sequence and structural conservation scores to produce the combined “Cons” score, in order to determine whether sequence-based alignment or structure-based alignment performed better in a given region. Clearly, this *ω*represents a possible tuning parameter, which a computational biologist aligning proteins known to be of closer or more remote homology might use to adjust the performance of Formatt. As the “Cons” score itself does not incorporate core length, it favors shorter, tighter alignments. One possible improvement could be to compute a score that trades off this score against core length, as in [8]. Another possible improvement would be to run all available sequence aligners on each region, and choose the best alignment from among all of them as well, as we now choose between one sequence aligner and the structural alignment. However, as MAFFT produces the shortest but best-scoring alignments, this would lead to nearly always preferring MAFFT over the other sequence aligners unless a modified score which incorporated core length were used.

Likewise, while Formatt performed similarly regardless of the choice of CLUSTALW, ProbCons, or MUSCLE as a sequence aligner, MAFFT produced distinctly shorter but more highly conserved alignments, and this distinction was magnified at the more remote level of homology exhibited by SABMARK’s twilight zone benchmark. Thus, a user may also prefer MAFFT for more closely homologous alignments, and MUSCLE for more remote homologs.

Benchmark alignments produced by Formatt with each of the four sequence aligners, as well as the Formatt software (under the GNU Public License) are available at http://bcb.cs.tufts.edu/formatt/.

## Competing interest

The authors declare that they have no competing interests.

## Authors’ contributions

ND, SN and LC conceived and designed the experiments. ND and SN performed the experiments. ND analyzed the data. ND and LC wrote the manuscript. All authors read and approved the final manuscript.

## References

[B1] Hasegawa H Holm L Advances and pitfalls of protein structural alignment Curr Opin Struct Biol 2009 19 3 341 348 10.1016/j.sbi.2009.04.003 19481444

[B2] Grishin NV KH domain: one motif, two folds Nucleic Acids Res 2001 29 3 638 643 10.1093/nar/29.3.638 11160884 PMC30387

[B3] O’Sullivan O Suhre K Abergel C Higgins D Notredame C 3DCoffee: combining protein sequences and structures with multiple sequence alignments J Mol Biol 2004 340 385 395 10.1016/j.jmb.2004.04.058 15201059

[B4] Pei J Kim BH Grishin NV PROMALS3D: a tool for multiple protein sequence and structure alignments Nucleic Acids Res 2008 36 2295 2300 10.1093/nar/gkn072 18287115 PMC2367709

[B5] Madhusudhan M Webb BM Marti-Renom MA Eswar N Sali A Alignment of multiple protein structures based on sequence and structure features Protein Engineering, Design and Selection 2009 22 569 574 10.1093/protein/gzp040 PMC2909824 19587024

[B6] Kim C Lee B Accuracy of structure-based sequence alignment of automatic methods BMC Bioinf 2007 8 355 10.1186/1471-2105-8-355 PMC2039753 17883866

[B7] Menke M Berger B Cowen L Matt: local flexibility aids protein multiple structure alignment PLoS Comput Biol 2008 4 e10 10.1371/journal.pcbi.0040010 18193941 PMC2186361

[B8] Daniels N Kumar A Cowen L Menke M Touring protein space with Matt Bioinf Res App 2010 6053/2010 18 28 http://www.springerlink.com/index/q9j12472213qtx28.pdf 10.1109/TCBB.2011.70 PMC3355523 21464511

[B9] Konagurthu A Whisstock J Stuckey P Lesk A MUSTANG: A multiple structural alignment algorithm Proteins: Structure, Function, and Bioinformatics 2006 64 559 574 10.1002/prot.20921 16736488

[B10] Notredame C Higgins D Heringa J T-Coffee: a novel method for fast and accurate multiple sequence alignment J Mol Biol 2000 302 205 217 10.1006/jmbi.2000.4042 10964570

[B11] VanWalle I Lasters I Wyns L SABmark–a benchmark for sequence alignment that covers the entire known fold space Bioinformatics 2005 21 1267 1268 10.1093/bioinformatics/bth493 15333456

[B12] Shatsky M Nussinov R Wolfson H Optimization of multiple-sequence alignment based on multiple-structure alignment Proteins: Structure, Function and Bioinformatics 2006 62 209 217 10.1002/prot.20665 16294339

[B13] Thompson JD Higgins DG Gibson TJ CLUSTAL-W: improving the sensitivity of progressive multiple sequence alignment through sequence weighting, position-specific gap penalties and weight matrix choice Nucleic Acids Res 1994 22 4673 4680 10.1093/nar/22.22.4673 7984417 PMC308517

[B14] Edgar R MUSCLE: multiple sequence alignment with high accuracy and high throughput Nucleic Acids Res 2004 32 1792 1797 10.1093/nar/gkh340 15034147 PMC390337

[B15] Do C Mahabhashyam M Brudno M Batzoglou S ProbCons: probabilistic consistency-based multiple sequence alignment Genome Res 2005 15 220 240 10.1101/gr.2821705 PMC546535 15687296

[B16] Katoh K Toh H Recent developments in the MAFFT multiple sequence alignment program Briefings in Bioinformatics 2008 9 286 298 10.1093/bib/bbn013 18372315

[B17] Mizuguchi K Deane C Blundell TL Overington J HOMSTRAD: a database of protein structure alignments for homologous families Protein Sci 1998 11 2469 2471 9828015 10.1002/pro.5560071126 PMC2143859

[B18] Kumar A Cowen L Recognition of beta structural motifs using hidden Markov models trained with simulated evolution Bioinformatics 2010 26 i287 i293 10.1093/bioinformatics/btq199 20529918 PMC2881384

[B19] Menke M Berger B Cowen L Markov random fields reveal an N-terminal double propeller motif as part of a bacterial hybrid two-component sensor system PNAS 2010 107 4069 4074 10.1073/pnas.0909950107 20147619 PMC2819974

[B20] Daniels NM Hosur R Berger B Cowen LJ SMURFLite: combining simplified Markov random fields with simulated evolution improves remote homology detection for beta-structural proteins into the twilight zone Bioinformatics 2012 28 9 1216 1222 10.1093/bioinformatics/bts110 22408192 PMC3338012

[B21] Shatsky M Nussinov R Wolfson H Flexible protein alignment and hinge detection Proteins 2002 48 242 256 10.1002/prot.10100 12112693

[B22] Ye Y Godzik A Flexible structure alignment by chaining aligned fragment pairs allowing twists Bioinformatics 2003 Suppl 2 II246 II255 14534198 10.1093/bioinformatics/btg1086

[B23] Ye Y Godzik A Multiple flexible structure alignment using partial order graphs Bioinformatics 2005 21 2362 2369 10.1093/bioinformatics/bti353 15746292

[B24] Mosca R Brannetti B Schneider TR Alignment of protein structures in the presence of domain motions BMC Bioinformatics 2008 9 352 10.1186/1471-2105-9-352 18727838 PMC2535786

[B25] Salem S Zaki MJ Bystroff C FlexSnap: Flexible non-sequential protein structure alignment Algorithms Mol Biol 2010 12 5 1 13 10.1186/1748-7188-5-12 PMC2846951 20047669

[B26] Eddy S Profile hidden Markov models Bioinformatics 1998 14 755 763 10.1093/bioinformatics/14.9.755 9918945

